# Effects of perioperative fluid management on postoperative outcomes in liver transplantation: a systematic review protocol

**DOI:** 10.1186/s13643-018-0841-3

**Published:** 2018-10-31

**Authors:** François Martin Carrier, Michaël Chassé, Han Ting Wang, Pierre Aslanian, Marc Bilodeau, Alexis F. Turgeon

**Affiliations:** 1 Department of Anesthesiology, Centre hospitalier de l’Université de Montréal (CHUM), 1000, rue St-Denis, 4e étage, Pavillon D, porte D04-5028, Montréal, Québec H2X 0C1 Canada; 2 Department of Medicine - Critical Care Division, Centre hospitalier de l’Université de Montréal (CHUM), 1000, rue St-Denis, 10e étage, Pavillon D, porte D10-2143, Montréal, Québec H2X 0C1 Canada; 3 Centre de recherche du CHUM (CRCHUM), 900, rue Saint-Denis, Montréal, Québec H2X 0A9 Canada; 4 Department of Medicine - Critical Care Division, CIUSSS de l’Est-de-l’île-de-Montréal - Hôpital Maisonneuve-Rosemont, 5415 Boulevard de l’Assomption, Montréal, Québec H1T 2M4 Canada; 5 Liver Unit, Centre hospitalier de l’Université de Montréal (CHUM), Université de Montréal, 1000, rue St-Denis, Montréal, Québec H2X 0C1 Canada; 6 Population Health and Optimal Health Practices Unit (Trauma - Emergency - Critical Care Medicine), CHU de Québec - Université Laval Research Center, Université Laval, 1401, 18e rue, Québec, Québec G1J 1Z4 Canada; 7 Department of Anesthesiology and Critical Care Medicine, Division of Critical Care Medicine, Université Laval, 1050 Avenue de la Médecine, Québec, Québec G1V 0A6 Canada

**Keywords:** Liver transplantation, Complications, Fluid management, Fluid resuscitation, Fluid administration, Goal-directed therapy, Phlebotomy, Restrictive fluid, Liberal fluid

## Abstract

**Background:**

Liver transplant recipients suffer many complications, but few intraoperative interventions supported by high-quality evidence have been found effective to reduce their incidence or severity. Fluid balance has been proposed as an important aspect of perioperative care in high-risk recipients. We will conduct a systematic review aimed at evaluating the effects of restrictive perioperative fluid management strategies compared to liberal ones on clinically significant postoperative outcomes.

**Methods:**

We will search through major databases (CINAHL Complete, EMB Reviews, EMBASE, MEDLINE, PubMed, and the gray literature (CADTH, Clinical Trials, National Guideline Clearing House, NICE, MedNar, Google Scholar and Open Grey)), from inception up to a date close to the review submission for publication, for eligible studies. Randomized controlled trials and comparative non-randomized studies (prospective or retrospective) comparing two fluid management strategies (or two outcomes with available data on fluid volume received for observational studies) on adult liver recipients will be included. Eligible studies will have to report at least one postoperative complication or mortality. Our primary outcome will be acute renal failure and our secondary exploratory outcomes will be all other postoperative complications and mortality. Study selection and data abstraction using an electronic standardized form will be performed by three authors. Risk of bias will be evaluated and data will be pooled if limited clinical diversity is observed.

**Discussion:**

Human organs available for transplantation are scarce resources. Strategies to improve recipients’ survival are needed. We hypothesize that restrictive fluid management strategies will be associated with better postoperative outcomes than liberal fluid management strategies. This systematic review will improve our understanding of the available evidence and help us better inform future clinical trials.

**Systematic review registration:**

This systematic review protocol is registered in PROSPERO (CRD42017054970).

**Electronic supplementary material:**

The online version of this article (10.1186/s13643-018-0841-3) contains supplementary material, which is available to authorized users.

## Background

Liver transplantation is the last line of therapy for severe end-stage liver disease and is increasingly performed throughout the world [1, 2]. In the past decade, overall waiting list and post-transplant survival rates have increased alongside postoperative complications, a significant burden for patient care [3, 4]. Due to the scarcity of human organs, strategies to improve recipients’ outcomes and organ survival are needed [2, 4].

Liver transplant recipients suffer, on average, more than three postoperative complications, with over half of them being severe [5, 6]. Several perioperative events and factors seem associated with the risk of complications [5, 7–17]. Among these, perioperative fluid management has been associated with postoperative complications and proposed as an important aspect of care in high-risk recipients [16, 18, 19]. A recent US survey revealed that more than 60% of anesthesiologists use either phlebotomy or normovolemic hemodilution to reduce blood transfusions and improve post-transplant outcomes, even though few of these interventions are supported by high-quality evidence [20, 21]. The impact of perioperative fluid balance on postoperative complications is better understood in other surgical populations, with hundreds of different combinations of fluid management protocols and hemodynamic goals studied over the past decade [22]. Perioperative fluid imbalance, defined as too little or too much fluid, was recently associated with a greater than 60% increase in postoperative complications after major abdominal surgery [23]. Recent systematic reviews suggest that cardiac output-guided fluid administration, compared to either fixed restrictive or fixed liberal strategies, reduces postoperative complications by 20–30% in patients undergoing major surgery [24, 25, 26], thus underscoring the significant role of fluid management in this population. More importantly, a recent multicenter clinical trial showed an increased incidence of acute renal failure when a fixed restrictive perioperative fluid strategy was compared to a liberal one during major abdominal surgery [27]. Liver transplantations were not included in any of these studies.

Since evidence suggests that specific perioperative fluid management strategies can improve or worsen postoperative outcomes in many surgical populations, that such strategies are being used in the liver transplant population without high-quality data, and that physicians are eager to use perioperative fluid and blood volume management strategies to improve outcomes in this population, the role of perioperative fluid management strategies in liver transplantation needs to be better understood [5, 7–16, 20, 21, 23–25, 26]. Therefore, we will conduct a systematic review aimed at evaluating the effects of a restrictive perioperative fluid management strategy compared to a liberal strategy on clinically significant outcomes in adult patients undergoing liver transplantation.

## Methods

### Study design

This systematic review will be conducted according to the standard methodology developed by the Cochrane Collaboration [28], and the results will be reported according to the PRISMA statement [29]. This protocol was designed according to the PRISMA-P statement (see Additional file 1 for associated checklist) [30].

### Research question

“What are the effects of a perioperative restrictive fluid management strategy, compared to a liberal one, on acute renal failure in adult patients undergoing liver transplantation?” We hypothesize that the use of restrictive fluid management strategies will be associated with a lower incidence of acute renal failure.

### Search strategy and information sources

We will search the bibliographic electronic databases CINAHL Complete (from 1937 onwards), EMB Reviews (from 1991 onwards), EMBASE (from 1974 onwards), MEDLINE (from 1946 onwards), PubMed (from inception), and the gray literature (CADTH, Clinical Trials, National Guideline Clearing House, NICE, MedNar, Google Scholar, and Open Grey, from inception) up to a date close to the review submission for publication. The search will incorporate words and expressions for two conceptual groups: *liver transplantation* and *fluid therapy*. For each database, we will use words and expressions from controlled vocabulary (MESH, EMTREE, and others) and free text searching. The search strategy, prepared by an information specialist, is detailed in Additional file 2. Snowballing method will also be used to identify other studies within the references of selected studies. Hand searching for relevant abstracts published in the annual meeting supplements of the following organizations will be performed for the years available on their website: American Association for the Study of Liver Diseases (AASLD), European Association for the Study of Liver (EASL), International Liver Transplantation Society (ILTS), American Society of Transplantation (AST), and European Society of Organ Transplantation (ESOT).

### Study eligibility

Studies will be eligible if they meet the following criteria and report any outcome of interest to our study.

#### Types of studies

Randomized controlled trials, quasi-randomized trials, and comparative non-randomized studies (prospective or retrospective) will be included. Randomized controlled trials will be considered if at least two different fluid management strategies are compared, one being more restrictive than the other, regardless of the methods used. Observational studies will be included if at least two groups with different fluid management strategies, or different amounts of fluid administered, are compared. Observational studies with outcome-based reported groups will also be included if the volume of fluid received can be extracted for each group. Editorials, letters, narrative type reviews, case reports, and animal studies will be excluded.

#### Participants

We will include studies if more than 80% of participants are adults (≥ 18 years old or as defined in individual studies) undergoing liver transplantation alone (deceased or living donor graft). We will exclude studies in which more than 20% of the participants received a combined transplantation (liver + kidney, liver + lung or liver + heart).

#### Intervention

The intervention group will be composed of patients receiving a restrictive perioperative fluid management strategy (applied in the intraoperative period, the postoperative period, or both). Any strategy or type of fluid management protocol that limits the amount of fluid administered by the clinician will be considered a restrictive fluid strategy (early goal-directed protocols, weight-based protocols, low-CVP protocols, phlebotomies, pre-determined volume management protocols, retrospectively classified received fluid strategy, etc.). In observational studies reporting groups with different volumes of fluid received, the group with the lowest volume will be considered the intervention group.

#### Comparator

The control group will be composed of patients receiving a more liberal perioperative fluid management strategy than the intervention group. Any type of fluid management protocol will be considered.

#### Outcome measures

Our main efficacy outcome is the incidence of acute renal failure (any definition and any time point before 30 postoperative days).

Our additional exploratory outcomes are as follows:

- Mortality (hospital and latest reported mortality).
- Perioperative complications including (but not restricted to): graft failure (any definition); biliary complications (leak, strictures, and/or ischemic cholangiopathy (any definition)); pulmonary complications (pneumonia, pulmonary edema, acute respiratory distress syndrome, duration of mechanical ventilation, ventilation-free days at latest); cardiovascular complications (myocardial infarction, arrhythmias, shock, thromboembolic events); infectious complications (wound infection, abdominal abscess, bacteremia, catheter-related bloodstream infection, urinary tract infection).
- Perioperative bleeding; postoperative red blood cell transfusions; postoperative procoagulant blood products;
- Intensive care unit (ICU) length of stay and ICU readmission; hospital length of stay.

Acute renal failure has been chosen as a primary outcome because of its high incidence following liver transplantation (13–71%), its association with intraoperative events, the burden on postoperative care and mortality, and because it is a patient-centered outcome [5, 6, 10, 11, 31, 32]. The worldwide low postoperative mortality in this population (10% at 1 year) is highly likely to preclude the observation of a treatment effect from different perioperative interventions [1, 33]. Mortality is, however, among our additional exploratory outcomes.

#### Year of publication and language

Eligibility will not be restricted by year nor by language of publication.

### Data management

#### Study selection

We will use the Endnote X8.2 software to merge retrieved titles, remove duplicates, and screen titles and abstracts. Three investigators will review every potentially eligible citation to identify all relevant studies (one investigator (FMC) will review all of them, and two others (PA, MC) will each review half of them). Two investigators (FMC, MC) will do a full-text review of selected citations to confirm study eligibility before extracting data. Disagreement will be resolved by consensus (FMC, MC, PA).

#### Data abstraction

Two investigators (FMC and HTW) will independently review every eligible study retrieved from the search and extract data with a standardized electronic data extraction form (see Additional file 3). Studies in languages other than English or French will be translated before abstraction. Data will be kept in a secured database. Disagreement will be resolved by consensus. Authors of retrieved articles will be contacted for additional information if deemed necessary, such as a potential available but not reported outcome.

### Data items

We will retrieve data on study characteristics, population characteristics, type of donor, perioperative period when the intervention is applied (and the associated volume of fluid received), type of intervention, type of fluid received, bleeding, transfusion of blood products, and every reported review outcome. Co-interventions such as the type of vasopressors used, the use of a coagulation management protocol, and transfusion thresholds will be also extracted.

### Risk of bias (ROB) assessment

ROB assessment will be performed at the study level in all important domains for both the study’s primary outcome and review’s primary outcome (acute renal failure). The effect of starting and adhering to the intervention will be assessed. Two investigators (FMC and HTW) will independently assess ROB. Disagreement will be resolved by consensus. ROB assessment for both outcomes, if available, will be reported for each individual study using a risk of bias summary figure and detailed information for each evaluated domain as a supplementary appendix.

The Cochrane ROB assessment tool 2.0 for randomized trials will be used for RCTs [34]. For observational studies, the ROBINS-I tool for non-randomized studies of interventions will be used and the following confounding domains that might influence selection of exposure (fluid management received) and the measure of effect (complications) will be included in our ROB assessment: comorbidities, baseline severity of the underlying liver disease, and baseline severity of non-liver organ failure [35]. Application of important co-interventions in both groups will also be included in the ROB assessment of observational studies, especially the use procoagulant blood products as prophylaxis and the use of a coagulation management protocol. Publication bias will be explored with a funnel plot using a common significant outcome.

### Data synthesis

#### Main analyses

We will summarily describe retrieved observational studies and randomized controlled trials separately. We will also report studies separately based on the timing of the intervention (intraoperative, postoperative, or both). We will detail study populations, fluid management protocols used, the type and amount of fluid, and blood products received and outcomes (with definition and time point used). In circumstances where pooling of studies is deemed inappropriate, we will only provide a qualitative discussion of the findings. If data pooling is appropriate based on low clinical diversity across studies (similar population and interventions), we will pool outcomes using Review Manager 5.3 (The Cochrane Collaboration, 2014). The measure of effect of dichotomous data will be risk ratios (RR) with 95% confidence intervals and mean differences (MD) with 95% confidence intervals for continuous data. We will use random effects models according to the DerSimonian-Laird method to take into account the underlying variation across studies. We will use the *I*^2^ measure of consistency across trials to evaluate heterogeneity and will report both this score and the Cochrane’s *Q* test [36].

#### Subgroup analyses

We will perform a series of subgroup analyses based on study characteristics to further explore our findings and explain potential sources of heterogeneity. We will investigate (1) the risk of bias (low vs. high risk of bias), (2) the difference in type of fluid management protocols (goal-directed therapy vs. other protocols), (3) the difference in the type of fluid received (synthetic colloid vs. not), (4) the difference in the use of a coagulation management protocol as a co-intervention (use vs. non-use), and (5) the type of graft used (living vs. cadaveric donor).

### Summary of findings

We will summarize our findings in a table generated by the GRADEpro web-based tool that will include the quality of cumulative evidence using GRADE criteria [37, 38]. All reported outcomes will be included.

## Discussion

Perioperative complications in liver transplant recipients are frequent, especially acute renal failure (13–71%), and are associated with postoperative mortality [5, 6, 10, 11]. Since human organs available for transplantation are a scarce resource, investigation of new perioperative strategies to improve recipients’ outcomes is required. There is a paucity of high-quality evidence reporting the impact of different intraoperative interventions to achieve these goals in the liver transplant population, and fluid management is an important part of perioperative care.

We expect to find a limited number of clinical trials and a moderate amount of observational studies with a moderate to serious risk of bias. The strength of this systematic review will be the broad evaluation of available evidence on perioperative fluid management strategies by including all studies reporting at least one clinically significant postoperative outcome. Our review will be limited by the potentially low methodological quality of the included studies, the possible clinical diversity between them, the probable insufficient power and information size to draw any definitive conclusions, and its exploratory nature. If outcome data allow performance of multiple meta-analyses, the use of multiple statistical testing might also limit the interpretation of findings. Our systematic review will, however, improve our understanding of the available evidence and inform future research in the field.

## Additional files

Additional file 1: PRISMA-P 2015 Checklist. (PDF 5108 kb)

Additional file 2: Search strategy. (DOCX 46 kb)

Additional file 3: Data extraction form. (DOCX 81 kb)
