# Supplementary material for: Effects of perioperative fluid management on postoperative outcomes in liver transplantation: a systematic review protocol
Source: Syst Rev. 2018 Oct 31;7:180. doi: 10.1186/s13643-018-0841-3 (PMC6211404; doi:10.1186/s13643-018-0841-3)
Supplement: Supplementary file 2 — Search strategy. (DOCX 46 kb) [file 13643_2018_841_MOESM2_ESM.docx]

**Additional file 2: Search strategy**

| Database | |
| --- | --- |
| Database | Ovid MEDLINE(R) Epub Ahead of Print, In-Process & Other Non-Indexed Citations, Ovid MEDLINE(R) Daily, Ovid MEDLINE and Versions(R) 1946 to July 05, 2018 |
| Interface | OvidSP |
| Research date | July 10, 2018 |
| Filters | - |

| Syntax | |
| --- | --- |
| / | Exact Subject Heading |
| */ | Focus on Exact Subject Heading |
| tw | Text word field in MEDLINE includes Title (TI) and Abstract (AB) |
| kw | Keywords |
| or, and | Boolean operators |
| adj2 | The Adjacent operator |
| * | Truncation |

| Search strategy |
| --- |

1 exp Fluid Therapy/ (18515)

2 exp Central Venous Pressure/ (4355)

3 exp Phlebotomy/ (5391)

4 (resuscitation adj2 strategy).kw,tw. (176)

5 (goal-directed adj2 (therap* or strategy)).tw,kw. (1185)

6 (volume adj2 resuscitation).tw,kw. (1134)

7 CVP.tw,kw. (2891)

8 (phlebotomy or liberal or restrictive).tw,kw. (33429)

9 (infus* adj2 (intraven* or parenter* or intraoss*)).tw,kw. (36322)

10 (fluid adj2 (replac* or restor* or administ* or manag* or imbalanc* or therap* or resuscitation or strategy or responsiveness or balanc* or crystalloid or parenteral or infusion or load* or overload*)).tw,kw. (23247)

11 (pulse adj2 contour$ analy*).tw,kw. (414)

12 picco.tw,kw. (439)

13 (stroke adj2 volume adj2 (varia* or change* or adjust* or alter*)).tw,kw. (1210)

14 (pulse adj2 pressure adj2 (varia* or change* or adjust* or alter*)).tw,kw. (817)

15 (#esophag* adj2 doppler).tw,kw. (194)

16 (pulse adj2 contour* analy*).tw,kw. (414)

**17 or/1-16 (117479)**

18 exp Liver Transplantation/ (51653)

19 ((liver or hepatic) adj2 (transplant* or graft* or allograft* or recipient*)).tw,kw. (58569)

**20 18 or 19 (68723)**

**21 17 and 20 (715)**

| Database | |
| --- | --- |
| Database | MEDLINE |
| Interface | PubMed |
| Research date | 10 July, 2018 |
| Filters |  |

| Syntax | |
| --- | --- |
| [MeSH Terms] | Medical Subject Heading |
| OR, AND | Boolean operators |
| * | Truncation |
| [tiab] | Title and abstract |
| [ot] | Oher term, includes the author’s key words |

| Search strategy |
| --- |

"Liver Transplantation"[Mesh] **OR** liver transplant*[tiab] **OR** hepatic transplant*[tiab] **OR** liver graft*[tiab] **OR** hepatic graft*[tiab] **OR** liver allograft*[tiab] **OR** hepatic recipient*[tiab] **OR** hepatic allograft*[tiab] **OR** liver recipient*[tiab] **OR** liver transplant*[ot] **OR** hepatic transplant*[ot] **OR** liver graft*[ot] **OR** hepatic graft*[ot] **OR** liver allograft*[ot] **OR** hepatic recipient*[ot] **OR** hepatic allograft*[ot] **OR** liver recipient*[ot]

**AND**

(((((((((((crystalloid fluid[Title/Abstract]) **OR** parenteral fluid restriction[Title/Abstract]) **OR** fluid infusion[Title/Abstract]) **OR** fluid load*[Title/Abstract]) **OR** fluid overload*[Title/Abstract]) **OR** crystalloid fluid[Other Term]) **OR** parenteral fluid restriction[Other Term]) **OR** fluid infusion[Other Term]) **OR** fluid load*[Other Term]) **OR** fluid overload*[Other Term])) **OR** ((((("Fluid Therapy"[MeSH Terms] **OR** fluid therap*[tiab] **OR** fluid therap*[ot] **OR** fluid resuscitation[tiab] **OR** fluid resuscitation[ot] **OR** fluid strategy[tiab] **OR** fluid strategy[ot] **OR** resuscitation strategy[tiab] **OR** resuscitation strategy[ot] **OR** goal-directed therap*[tiab] **OR** goal-directed therap*[ot] **OR** goal-directed strategy[tiab] **OR** goal-directed strategy[ot] **OR** volume resuscitation[tiab] **OR** volume resuscitation[ot] **OR** CVP[tiab] **OR** CVP[ot] **OR** "Central venous pressure"[MeSH Terms] **OR** phlebotomy[tiab] **OR** phlebotomy[ot] **OR** "Phlebotomy"[MeSH Terms] **OR** liberal[tiab] **OR** liberal[ot] **OR** restrictive[tiab] **OR** restrictive[ot] **OR** fluid responsiveness[tiab] **OR** fluid responsiveness[ot]))) **OR** (((((((((((((((((((((((stroke pressure alter*[Other Term]) **OR** stroke pressure adjust*[Other Term]) **OR** stroke pressure change*[Other Term]) **OR** stroke pressure varia*[Other Term]) **OR** stroke volume alter*[Other Term]) **OR** stroke volume adjust*[Other Term]) **OR** stroke volume change*[Other Term]) **OR** stroke volume varia*[Other Term]) **OR** picco[Other Term]) **OR** esophag* doppler[Other Term]) **OR** pulse contour analy*[Other Term]) **OR** fluid* imbalanc*[Other Term]) **OR** fluid* balanc*[Other Term]) **OR** fluid* manag*[Other Term]) **OR** fluid* administ*[Other Term]) **OR** fluid* restor*[Other Term]) **OR** fluid* replac*[Other Term]) **OR** intraoss* infus*[Other Term]) **OR** parenter* infus*[Other Term]) **OR** intraven* infus*[Other Term]))) **OR** (((((((((((((((((((((stroke pressure alter*[Title/Abstract]) **OR** stroke pressure adjust*[Title/Abstract]) **OR** stroke pressure change*[Title/Abstract]) **OR** stroke pressure varia*[Title/Abstract]) **OR** stroke volume alter*[Title/Abstract]) **OR** stroke volume adjust*[Title/Abstract]) **OR** stroke volume change*[Title/Abstract]) **OR** stroke volume varia*[Title/Abstract]) **OR** picco[Title/Abstract]) **OR** esophag* doppler[Title/Abstract]) **OR** pulse contour analy*[Title/Abstract]) **OR** fluid* imbalanc*[Title/Abstract]) **OR** fluid* balanc*[Title/Abstract]) **OR** fluid* manag*[Title/Abstract]) **OR** fluid* administ*[Title/Abstract]) **OR** fluid* restor*[Title/Abstract]) **OR** fluid* replac*[Title/Abstract]) **OR** intraoss* infus*[Title/Abstract]) **OR** parenter* infus*[Title/Abstract]) **OR** intraven* infus*[Title/Abstract])))))

Total: 837 references

| Database | |
| --- | --- |
| Database | Embase 1974 to 2018 July 09 |
| Interface | OvidSP |
| Research date | July 10, 2018 |
| Filters |  |

| Syntax | |
| --- | --- |
| / | Exact Subject Heading |
| */ | Focus on Exact Subject Heading |
| tw | Text word field in EMBASE includes Title (TI), Abstract (AB) and Drug Trade Name (TN). |
| kw | Keywords |
| or, and | Boolean operators |
| adj2 | The Adjacent operator |
| * | Truncation |

| Search strategy |
| --- |

1 fluid therapy/ (19341)

2 central venous pressure/ (11981)

3 phlebotomy/ (10002)

4 (resuscitation adj2 strategy).kw,tw. (256)

5 (goal-directed adj2 (therap* or strategy)).tw,kw. (2098)

6 (volume adj2 resuscitation).tw,kw. (1701)

7 CVP.tw,kw. (5205)

8 (phlebotomy or liberal or restrictive).tw,kw. (46842)

9 (infus* adj2 (intraven* or parenter* or intraoss*)).tw,kw. (45407)

10 (fluid adj2 (replac* or restor* or administ* or manag* or imbalanc* or therap* or resuscitation or strategy or responsiveness or balanc* or crystalloid or parenteral or infusion or load* or overload*)).tw,kw. (34757)

11 (pulse adj2 contour$ analy*).tw,kw. (719)

12 picco.tw,kw. (938)

13 (stroke adj2 volume adj2 (varia* or change* or adjust* or alter*)).tw,kw. (1781)

14 (pulse adj2 pressure adj2 (varia* or change* or adjust* or alter*)).tw,kw. (1254)

15 (#esophag* adj2 doppler).tw,kw. (279)

16 (pulse adj2 contour* analy*).tw,kw. (719)

**17 or/1-16 (162509)**

18 liver transplantation/ (92609)

19 ((liver or hepatic) adj2 (transplant* or graft* or allograft* or recipient*)).tw,kw. (95183)

**20 18 or 19 (114158)**

**21 17 and 20 (1564)**

**22 limit 21 to embase (960)**

| Database | |
| --- | --- |
| Databases | EBM Reviews - Cochrane Database of Systematic Reviews 2005 to July 5, 2018,  EBM Reviews - ACP Journal Club 1991 to June 2018,  EBM Reviews - Database of Abstracts of Reviews of Effects 1st Quarter 2016,  EBM Reviews - Cochrane Clinical Answers June 2018,  EBM Reviews - Cochrane Central Register of Controlled Trials June 2018,  EBM Reviews - Cochrane Methodology Register 3rd Quarter 2012,  EBM Reviews - Health Technology Assessment 4th Quarter 2016,  EBM Reviews - NHS Economic Evaluation Database 1st Quarter 2016 |
| Interface | OvidSP |
| Research date | July 10, 2018 |
| Filters | - |

| Syntax | |
| --- | --- |
| / | Exact Subject Heading |
| kw | Keywords |
| af | All fields |
| or, and | Boolean operators |
| * | Truncation |
| adj2 | The Adjacent operator |

| Search strategy |
| --- |

1 exp Fluid Therapy/ (1506)

2 exp Central Venous Pressure/ (230)

3 exp Phlebotomy/ (398)

4 (resuscitation adj2 strategy).kw,tw. (55)

5 (goal-directed adj2 (therap* or strategy)).tw,kw. (401)

6 (volume adj2 resuscitation).tw,kw. (165)

7 CVP.tw,kw. (711)

8 (phlebotomy or liberal or restrictive).tw,kw. (2603)

9 (infus* adj2 (intraven* or parenter* or intraoss*)).tw,kw. (8994)

10 (fluid adj2 (replac* or restor* or administ* or manag* or imbalanc* or therap* or resuscitation or strategy or responsiveness or balanc* or crystalloid or parenteral or infusion or load* or overload*)).tw,kw. (4556)

11 (pulse adj2 contour$ analy*).tw,kw. (67)

12 picco.tw,kw. (96)

13 (stroke adj2 volume adj2 (varia* or change* or adjust* or alter*)).tw,kw. (275)

14 (pulse adj2 pressure adj2 (varia* or change* or adjust* or alter*)).tw,kw. (376)

15 (#esophag* adj2 doppler).tw,kw. (56)

16 (pulse adj2 contour* analy*).tw,kw. (67)

**17 or/1-16 (18323)**

18 exp Liver Transplantation/ (1226)

19 ((liver or hepatic) adj2 (transplant* or graft* or allograft* or recipient*)).tw,kw. (4089)

**20 18 or 19 (4173)**

**21 17 and 20 (129)**

| **Database** | |
| --- | --- |
| Databases | CINAHL Complete |
| Interface | EBSCO |
| Research date | July 10, 2018 |
| Filters | - |

| **Syntax** | |
| --- | --- |
| MH | Exact Subject Headings |
| TI | Title |
| AB | Abstract |
| S (1, 2, 3…) | Search |
| OR, AND | Boolean operators |

| **Search strategy** |
| --- |

| **#** | **Question** | **Résultats** |
| --- | --- | --- |
| S1 | (MH "Liver Transplantation") OR ( ((liver or hepatic) N2 (transplant* or graft* or allograft* or recipient*)) ) | 7043 |
| S2 | ( (MH "Fluid Therapy") OR (MH "Fluid Therapy (Saba CCC)") OR (MH "Central Venous Pressure") OR (MH "Phlebotomy") ) OR ( (fluid N2 (replac* or restor* or administ* or manag* or imbalanc* or therap* or resuscitation or strategy or responsiveness or balanc* or crystalloid or parenteral or infusion or load* or overload*)) ) OR (resuscitation N2 strategy) OR ( (goal-directed N2 (therap* or strategy)) ) OR (volume N2 resuscitation) OR (CVP) OR ( (phlebotomy or liberal or restrictive) ) | 24581 |
| S3 | (infus* N2 (intraven* or parenter* or intraoss*)) | 12726 |
| S4 | (pulse N2 contour$ analy*) OR picco | 226 |
| S5 | ( (stroke N2 volume N2 (varia* or change* or adjust* or alter*)) ) OR ( (pulse N2 pressure N2 (varia* or change* or adjust* or alter*)) ) OR (#esophag* N2 doppler) OR (pulse N2 contour* analy*) | 789 |
| **S6** | **S2 OR S3 OR S4 OR S5** | 37084 |
| **S7** | **S1 AND S6** | 113 |
