# Supplementary material for: Effects of perioperative fluid management on postoperative outcomes in liver transplantation: a systematic review protocol
Source: Syst Rev. 2018 Oct 31;7:180. doi: 10.1186/s13643-018-0841-3 (PMC6211404; doi:10.1186/s13643-018-0841-3)
Supplement: Supplementary file 3 — Data extraction form. (DOCX 81 kb) [file 13643_2018_841_MOESM3_ESM.docx]

**Additional file 3: Data extraction form**

Reviewer’s initials: _________________________

Date: _____________________________________

**Section 1: Study identification**

Study ID: ____________________________

Study title: ________________________________________________________________________

________________________________________________________________________

Authors: ________________________________________________________________

Source: _________________________________________________________________

Year: ___________________ Volume: ____________________ Page:_______________

Country of origin: _________________________ Language:_______________________

Sources of funding: _______________________________________________________

Conflict of interest: ­­­­­­­_______________________________________________________

Comments:

**Section 2: Eligibility criteria**

- 1.Randomized controlled trial or observational cohort study (prospective or retrospective)
- 2. Adult patients (> 80%)
- 3. Patients in the cohort is undergoing a liver transplantation (> 80% of the participants)
- 4. More than 80% of the participants undergo a liver transplantation alone
- 5. Comparison of two fluid resuscitation strategies, one being more restrictive than the other
- 6. One of the following outcomes is reported:
  - mortality (any)
  - any post-operative complication among the followings:
    - acute renal failure,
    - hepatocellular insufficiency (graft failure),
    - biliary complications,
    - respiratory complications,
    - cardiovascular complications,
    - Infectious complications.
  - Other post-operative outcomes:
    - perioperative bleeding;
    - intra-operative and post-operative red blood cell transfusion;
    - intra-operative and post-operative hemostatic blood products transfusion;
    - ICU length of stay and ICU readmission;
    - Hospital length of stay.

**Section 3: Study characteristics**

Type of document:

- Journal publication
- Abstract
- Editorial
- Letter to the editor
- Book chapter
- Conference proceedings

| Type of study:  Experimental   - Randomized controlled trial - Quasi-randomized controlled trial   Observational   - Non-randomized prospective cohort study with a prospective comparator - Non-randomized prospective cohort study with a retrospective comparator - Non-randomized retrospective cohort study with a prospective comparator - Non-randomized retrospective cohort study with a retrospective comparator   Review   - Systematic review - Narrative review |
| --- |

Start date: ____________________________ End date: __________________________

Length of follow up: _______________________________________________________

Inclusion criteria:

1.______________________________________________________________________2.______________________________________________________________________3.______________________________________________________________________4.______________________________________________________________________5.______________________________________________________________________6.______________________________________________________________________

Exclusion criteria:

1.______________________________________________________________________2.______________________________________________________________________3.______________________________________________________________________4.______________________________________________________________________5.______________________________________________________________________6.______________________________________________________________________

Number of patients:

Screened: ______________________________________

Excluded: ______________________________________ ☐ Not specified

Included: _______________________________________

Detailed justification? ☐ Yes ☐ No ☐ Unclear

Number of participants lost to follow-up (overall and by group):____________________

☐ Not specified

Detailed justification ? ☐ Yes ☐ No ☐ Unclear

Presentation via flow diagram? ☐Yes ☐No ☐Unclear

**Section 4: Population characteristics**

| *Variable* | All patients | Group 1 (restrictive) | Group 2  (liberal) |
| --- | --- | --- | --- |
| **General characteristics** | | | |
| Patients analysed (n) |  |  |  |
| Gender (% male) |  |  |  |
| Age (mean ± SD) |  |  |  |
| Preoperative ward hospitalization (n) |  |  |  |
| Preoperative ICU hospitalization (n) |  |  |  |
| Previous abdominal surgery (n) |  |  |  |
| **Pre-operative liver failure score** | | | |
| CHILD-PUGH score (median, IQR) |  |  |  |
| MELD (mean ± SD) |  |  |  |
| MELD-Na (mean ± SD) |  |  |  |
|  |  |  |  |
| **Liver transplantation indication** | | | |
| Acute liver failure (n) |  |  |  |
| Cirrhosis (n) |  |  |  |
| Alcohol (n) |  |  |  |
| HCV (n) |  |  |  |
| HBV (n) |  |  |  |
| NASH (n) |  |  |  |
| CHC (n) |  |  |  |
| Auto-immune disease (n) |  |  |  |
| Wilson (n) |  |  |  |
| Other (n) |  |  |  |
| Retransplantation (n) |  |  |  |
| **Pre-operative organ failure and organ support** | | | |
| Preoperative renal failure (n) |  |  |  |
| Renal replacement therapy (n) |  |  |  |
| Albumin dialysis (n) |  |  |  |
| Vasopressors (n) |  |  |  |
| Mechanical ventilation (n) |  |  |  |
| **Pre-operative lab values** | | | |
| Bilirubin (mean ± SD) |  |  |  |
| Creatinine (mean ± SD) |  |  |  |
| INR (mean ± SD) |  |  |  |
| Na (mean ± SD) |  |  |  |
| Albumin (mean ± SD) |  |  |  |
| Hemoglobin (mean ± SD) |  |  |  |

**Section 5: Characteristics of the main interventions**

| Was the fluid management protocol applied intra-operatively? | YES | NO |
| --- | --- | --- |
| Was the fluid management protocol applied post-operatively? | YES | NO |
| Was the fluid management protocol different at different steps of the procedure (dissection, anhepatic, reperfusion, post-operative)?  *If YES, use appendix IIb for extracting supplementary data* | YES | NO |

**Group 1: Restrictive strategy**

- Pre-anhepatic phlebotomies
- Low-CVP management
- Weight-based restrictive fluid regimen (mL/kg/h)
- Goal-directed therapy (restrictive) (if yes, select the chosen goal):
  - Cardiac output: ___________
  - Pulse pressure variation: __________
  - Stroke volume variation: __________
  - Other: ________________________
- Usual care
- Retrospectively classified restrictive group
- Other restrictive fluid regimen

Additional information on fluid administration protocol: ________________________________________________________________________

**Group 2: Liberal strategy**

- Weight-based liberal fluid regimen (mL/kg/h)
- Goal-directed therapy (liberal) (if yes, select the chosen goal):
  - Cardiac output: ___________
  - Pulse pressure variation: __________
  - Stroke volume variation: __________
  - Other: ________________________
- Usual care
- Retrospectively classified liberal group
- Other liberal fluid regimen

Additional information on fluid administration protocol: ________________________________________________________________________

**Interventions at different steps of the procedure:**

Specify the fluid management protocol at each step in each group, if available:

Group 1: Restrictive strategy

1. Dissection: _________________
2. Anhepatic: _________________
3. Reperfusion: ________________
4. Post-operative: ______________

Group 2: Liberal strategy

1. Dissection: _________________
2. Anhepatic: _________________
3. Reperfusion: ________________
4. Post-operative: ______________

**Section 6: Co-interventions**

Fluids:

| Was the same fluids used in both groups? | YES | NO |
| --- | --- | --- |

If yes, what fluids were used for fluid resuscitation:

- NaCl 0,9%
- Balanced cristalloid (Ringer’s Lactate, Plasmalyte, Normosol)
- Starchs (Pentastarch, Tetrastarchs)
- Gelatins
- Isotonic albumin (4-5%)
- Hypertonic albumin (20-25%)
- Fresh frozen plasma

If no, specify fluids used in different groups:

Restrictive group:

- NaCl 0,9%
- Balanced cristalloid (Ringer’s Lactate, Plasmalyte, Normosol)
- Starchs (Pentastarch, Tetrastarchs)
- Gelatins
- Isotonic albumin (4-5%)
- Hypertonic albumin (20-25%)
- Fresh frozen plasma

Liberal group:

- NaCl 0,9%
- Balanced cristalloid (Ringer’s Lactate, Plasmalyte, Normosol)
- Starchs (Pentastarch, Tetrastarchs)
- Gelatins
- Isotonic albumin (4-5%)
- Hypertonic albumin (20-25%)
- Fresh frozen plasma

Transfusions and coagulation management:

| Was a transfusion threshold used in this study? | YES | NO |
| --- | --- | --- |
| If yes, was the transfusion threshold the same in both groups? | YES | NO |

If yes, red blood cells transfusion threshold: ­­­­­__________

If no, red blood cells transfusion threshold:

- Restrictive strategy group: __________
- Liberal strategy group: __________

| Was there prophylaxis use of FFP to correct INR? | YES | NO |
| --- | --- | --- |
| Was there a coagulation management protocol used? | YES | NO |
| If yes, was it used in both groups? | YES | NO |

If yes, on what monitoring tool was the protocol based:

- Central laboratory-based classical coagulation time
- Point-of-care classical coagulation time
- Central laboratory-based Thromboelastography/ Thromboelastometry
- Point-of-care Thromboelastography/ Thromboelastometry
- Other point-of-care coagulation management system

If no, specify monitoring tool used in different groups:

Restrictive group:

- Central laboratory-based classical coagulation time
- Point-of-care classical coagulation time
- Central laboratory-based Thromboelastography/ Thromboelastometry
- Point-of-care Thromboelastography/ Thromboelastometry
- Other point-of-care coagulation management system

Liberal group:

- Central laboratory-based classical coagulation time
- Point-of-care classical coagulation time
- Central laboratory-based Thromboelastography/ Thromboelastometry
- Point-of-care Thromboelastography/ Thromboelastometry
- Other point-of-care coagulation management system

Vasopressors:

| Were the same vasopressors used in both groups? | YES | NO |
| --- | --- | --- |

If yes, main vasopressors used:

- Phenylephrine
- Norepinephrine
- Vasopressin
- Terlipressin
- Other: _________

If different between groups:

Restrictive group:

- Phenylephrine
- Norepinephrine
- Vasopressin
- Terlipressin
- Other: _________

Liberal group:

- Phenylephrine
- Norepinephrine
- Vasopressin
- Terlipressin
- Other: _________

Other co-interventions (specify if applied in both groups):

________________________________________________________________________

**Section 7: Amount of intervention (fluid) received**

*If the amount of fluid or transfusion received are available for each step of the procedure, please use appendix IIb to substitute section 7.*

Amount of fluid received per group:

*Please indicate until when the postoperative fluid was calculated (hours or days): __________________*

| *Outcome* | Group 1 (restrictive) | Group 2  (liberal) | Mean/additional information |
| --- | --- | --- | --- |
| **Total fluid** | | | |
| Overall |  |  |  |
| Intraoperative - total |  |  |  |
| Postoperative |  |  |  |
| **Cristalloids** | | | |
| Overall |  |  |  |
| Intraoperative - total |  |  |  |
| Postoperative |  |  |  |
| **Synthetic colloids** | | | |
| Overall |  |  |  |
| Intraoperative - total |  |  |  |
| Postoperative |  |  |  |
| **Albumin** | | | |
| Overall |  |  |  |
| Intraoperative - total |  |  |  |
| Postoperative |  |  |  |

Transfusions received per group:

| *Outcome* | Group 1 (restrictive) | Group 2  (liberal) | Mean/additional information |
| --- | --- | --- | --- |
| **Red blood cell transfusions** | | | |
| Overall |  |  |  |
| Intraoperative - total |  |  |  |
| Postoperative |  |  |  |
| **Cell saver (reperfused)** | | | |
| Overall |  |  |  |
| Intraoperative - total |  |  |  |
| Postoperative |  |  |  |
| **Platelets** | | | |
| Overall |  |  |  |
| Intraoperative - total |  |  |  |
| Postoperative |  |  |  |
| **Fresh frozen plasma** | | | |
| Overall |  |  |  |
| Intraoperative - total |  |  |  |
| Postoperative |  |  |  |
| **Cryoprecipitates** | | | |
| Overall |  |  |  |
| Intraoperative - total |  |  |  |
| Postoperative |  |  |  |
| **Fibrinogen** | | | |
| Overall |  |  |  |
| Intraoperative - total |  |  |  |
| Postoperative |  |  |  |
| **Other blood products: ___________________** | | | |
| Overall |  |  |  |
| Intraoperative - total |  |  |  |
| Postoperative |  |  |  |
| **Other blood products: ___________________** | | | |
| Overall |  |  |  |
| Intraoperative - total |  |  |  |
| Postoperative |  |  |  |

Notes:

**Section 8: Outcomes**

*Please indicate in the right margin the outcome censoring time (7 days, 28 days, etc.).*

**Primary outcome**

*Please indicate the main outcome with a *.*

| *Outcome* | Group 1 (restrictive) | Group 2  (liberal) | Total | Censoring |
| --- | --- | --- | --- | --- |
| Acute renal failure |  |  |  |  |
| Acute renal failure requiring dialysis |  |  |  |  |

**Secondary outcomes**

*Please indicate the main outcome with a *.*

| *Outcome* | Group 1 (restrictive) | Group 2  (liberal) | Total | Censoring |
| --- | --- | --- | --- | --- |
| **Mortality** | | | | |
| Hospital mortality |  |  |  |  |
| Latest mortality |  |  |  |  |
| **Graft failure** | | | | |
| Graft failure |  |  |  |  |
| Graft failure requiring retransplantation |  |  |  |  |
| **Biliary complications** | | | | |
| Total biliary complications^+^ |  |  |  |  |
| Hospital |  |  |  |  |
| Latest |  |  |  |  |
| **Pulmonary complications (hospital)** | | | | |
| Pulmonary complications |  |  |  |  |
| Pneumonia |  |  |  |  |
| Pulmonary oedema - ARDS |  |  |  |  |
| Ventilator-free days at latest |  |  |  |  |
| **Cardiovascular complications (hospital)** | | | | |
| Cardiovascular complications |  |  |  |  |
| Myocardial infarction |  |  |  |  |
| Arythmias |  |  |  |  |
| Post-operative shock |  |  |  |  |
| Thrombo-embolic complications |  |  |  |  |
| **Infectious complications (hospital)** | | | | |
| Infectious complications |  |  |  |  |
| Wound complications |  |  |  |  |
| Abdominal abcess |  |  |  |  |
| Catheter-related bloodstream infection |  |  |  |  |
| Bacteriemia |  |  |  |  |
| Urinary tract infection |  |  |  |  |
| **Other complication outcomes** | | | | |
| Dindo-Clavien grade III and more complications |  |  |  |  |

+ Defined as biliary leak, anastomotic biliary stricture or ischemic cholangiopathy (intra-hepatic non-anastomotic strictures associated with either an abnormal biochemical cholestatic profile, a bacterial cholangitis, a hepatic abscess or a retransplantation).

**Other outcomes**

| *Outcome* | Group 1 (restrictive) | Group 2  (liberal) | Total | Censoring |
| --- | --- | --- | --- | --- |
| **Bleeding** | | | | |
| Overall |  |  |  |  |
| Intraoperative - total |  |  |  |  |
| Postoperative |  |  |  |  |
| **Length of stay** | | | | |
| Duration of ICU stay |  |  |  |  |
| ICU free days at 28 days |  |  |  |  |
| Duration of hospital stay |  |  |  |  |
| ICU readmission |  |  |  |  |

**Section 9: Risk of bias assessment**

9.1 Randomized controlled trials (*based on Cochrane Collaboration tool for assessing risk of bias*)

| **Domain** | **Support for judgement** | **Review authors’ judgement** |
| --- | --- | --- |
| *Selection bias.* |  |  |
| **Random sequence generation.** | Describe the method used to generate the allocation sequence in sufficient detail to allow an assessment of whether it should produce comparable groups. | - Low risk of bias - High risk of bias - Unclear risk |
| **Allocation concealment.** | Describe the method used to conceal the allocation sequence in sufficient detail to determine whether intervention allocations could have been foreseen in advance of, or during, enrolment. | - Low risk of bias - High risk of bias - Unclear risk |
| *Performance bias.* |  |  |
| **Blinding of participants and personnel (***Assessments should be made for each main outcome (or class of outcomes)).* | Describe all measures used, if any, to blind study participants and personnel from knowledge of which intervention a participant received. Provide any information relating to whether the intended blinding was effective. | - Low risk of bias - High risk of bias - Unclear risk |
| *Detection bias.* |  |  |
| **Blinding of outcome assessment** (*Assessments should be made for each main outcome (or class of outcomes))*. | Describe all measures used, if any, to blind outcome assessors from knowledge of which intervention a participant received. Provide any information relating to whether the intended blinding was effective. | - Low risk of bias - High risk of bias - Unclear risk |
| *Attrition bias.* |  |  |
| **Incomplete outcome data (***Assessments should be made for each main outcome (or class of outcomes)).* | Describe the completeness of outcome data for each main outcome, including attrition and exclusions from the analysis. State whether attrition and exclusions were reported, the numbers in each intervention group (compared with total randomized participants), reasons for attrition/exclusions where reported, and any re-inclusions in analyses performed by the review authors. | - Low risk of bias - High risk of bias - Unclear risk |
| *Reporting bias.* |  |  |
| **Selective reporting.** | State how the possibility of selective outcome reporting was examined by the review authors, and what was found. | - Low risk of bias - High risk of bias - Unclear risk |
| *Other bias.* |  |  |
| **Other sources of bias.** | State any important concerns about bias not addressed in the other domains in the tool.  If particular questions/entries were pre-specified in the review’s protocol, responses should be provided for each question/entry. | - Low risk of bias - High risk of bias - Unclear risk |

9.2 Non-randomized trials (*adapted from ROBINS-I tool for ROB assessment in non-randomised trials*)

***Please fill a “Section 9.2” for review’s primary outcome and individual study’s primary outcome if different.***

##

## **Specify a target randomized trial specific to the study**

| Design | Individually randomized / Cluster randomized / Matched (e.g. cross-over) |
| --- | --- |
| Participants |  |
| Experimental intervention |  |
| Comparator |  |

## **Is your aim for this study…?**

| □ | to assess the effect of *assignment to* intervention |
| --- | --- |
| □ | to assess the effect of *starting and adhering to* intervention |

## **Specify the outcome**

Specify which outcome is being assessed for risk of bias (typically from among those earmarked for the Summary of Findings table). Specify whether this is a proposed benefit or harm of intervention.

|  |
| --- |

## **Specify the numerical result being assessed**

In case of multiple alternative analyses being presented, specify the numeric result (e.g. RR = 1.52 (95% CI 0.83 to 2.77) and/or a reference (e.g. to a table, figure or paragraph) that uniquely defines the result being assessed.

|  |
| --- |

## **Preliminary consideration of confounders**

## Complete a row for each important confounding domain (i) listed in the review protocol; and (ii) relevant to the setting of this particular study, or which the study authors identified as potentially important.

## *A confounding domain is a pre-intervention prognostic factor that predicts whether an individual receives one or the other intervention of interest. “Important” confounding domains are those for which, in the context of this study, adjustment is expected to lead to a clinically important change in the estimated effect of the intervention. “Validity” refers to whether the confounding variable or variables fully measure the domain, while “reliability” refers to the precision of the measurement (more measurement error means less reliability).*

| **(i) Confounding domains listed in the review protocol** | | | | |
| --- | --- | --- | --- | --- |
| Confounding domain | Measured variable(s) | Is there evidence that controlling for this variable was unnecessary?* | Is the confounding domain measured validly and reliably by this variable (or these variables)? | Is failure to adjust for this variable (alone) expected to favour the experimental intervention or the comparator? |
| Comorbidities | Chronic renal failure  Anemia |  | - Yes - No - No information | - Favour experimental - Favour comparator - No information |
| Severity of disease | MELD score |  | - Yes - No - No information | - Favour experimental - Favour comparator - No information |
| Severe organ dysfunction | % of patients on RRT  % of patients on pressors  % of patients on MV |  | - Yes - No - No information | - Favour experimental - Favour comparator - No information |
| Bleeding | Intraoperative blood lost  (time-varying confounder - if fluid resuscitation strategy changes) |  | - Yes - No - No information | - Favour experimental - Favour comparator - No information |
|  |  |  | - Yes - No - No information | - Favour experimental - Favour comparator - No information |
|  |  |  |  |  |

| **(ii) Additional confounding domains relevant to the setting of this particular study, or which the study authors identified as important** | | | | |
| --- | --- | --- | --- | --- |
| Confounding domain | Measured variable(s) | Is there evidence that controlling for this variable was unnecessary?* | Is the confounding domain measured validly and reliably by this variable (or these variables)? | Is failure to adjust for this variable (alone) expected to favour the experimental intervention or the comparator? |
|  |  |  | - Yes - No - No information | - Favour experimental - Favour comparator - No information |
|  |  |  | - Yes - No - No information | - Favour experimental - Favour comparator - No information |
|  |  |  | - Yes - No - No information | - Favour experimental - Favour comparator - No information |
|  |  |  | - Yes - No - No information | - Favour experimental - Favour comparator - No information |
|  |  |  |  |  |

* In the context of a particular study, variables can be demonstrated not to be confounders and so not included in the analysis: (a) if they are not predictive of the outcome; (b) if they are not predictive of intervention; or (c) because adjustment makes no or minimal difference to the estimated effect of the primary parameter. Note that “no statistically significant association” is not the same as “not predictive”.

## **Preliminary consideration of co-interventions**

Complete a row for each important co-intervention (i) listed in the review protocol; and (ii) relevant to the setting of this particular study, or which the study authors identified as important.

## *“Important” co-interventions are those for which, in the context of this study, adjustment is expected to lead to a clinically important change in the estimated effect of the intervention.*

| **(i) Co-interventions listed in the review protocol** | | |
| --- | --- | --- |
| Co-intervention | Is there evidence that controlling for this co-intervention was unnecessary (e.g. because it was not administered)? | Is presence of this co-intervention likely to favour outcomes in the experimental intervention or the comparator |
| Use of blood products other than red blood cells for bleeding prophylaxis |  | Favour experimental / Favour comparator / No information |
| Use of a coagulation management protocol |  | Favour experimental / Favour comparator / No information |
|  |  | Favour experimental / Favour comparator / No information |
|  |  | Favour experimental / Favour comparator / No information |

| **(ii) Additional co-interventions relevant to the setting of this particular study, or which the study authors identified as important** | | |
| --- | --- | --- |
| Co-intervention | Is there evidence that controlling for this co-intervention was unnecessary (e.g. because it was not administered)? | Is presence of this co-intervention likely to favour outcomes in the experimental intervention or the comparator |
|  |  | Favour experimental / Favour comparator / No information |
|  |  | Favour experimental / Favour comparator / No information |

## **Risk of bias assessment**

Responses underlined in green are potential markers for low risk of bias, and responses in red are potential markers for a risk of bias. Where questions relate only to sign posts to other questions, no formatting is used.

|  | **Signalling questions** | **Description** | **Response options** |
| --- | --- | --- | --- |
| **Bias due to confounding** | | | |
| 1.1 Is there potential for confounding of the effect of intervention in this study?  **If N/PN to 1.1:** the study can be considered to be at low risk of bias due to confounding and no further signalling questions need be considered | |  | Y / PY / PN / N |
| **If Y/PY to 1.1**: determine whether there is a need to assess time-varying confounding: | |  |  |
| 1.2. Was the analysis based on splitting participants’ follow up time according to intervention received?  **If N/PN**, answer questions relating to baseline confounding (1.4 to 1.6)  **If Y/PY**, go to question 1.3. | |  | NA / Y / PY / PN / N / NI |
| 1.3. Were intervention discontinuations or switches likely to be related to factors that are prognostic for the outcome?  **If N/PN**, answer questions relating to baseline confounding (1.4 to 1.6)  **If Y/PY**, answer questions relating to both baseline and time-varying confounding (1.7 and 1.8) | |  | NA / Y / PY / PN / N / NI |

| **Questions relating to baseline confounding only** |  |  |
| --- | --- | --- |
| 1.4. Did the authors use an appropriate analysis method that controlled for all the important confounding domains? |  | NA / Y / PY / PN / N / NI |
| 1.5. **If Y/PY to 1.4**: Were confounding domains that were controlled for measured validly and reliably by the variables available in this study? |  | NA / Y / PY / PN / N / NI |
| 1.6. Did the authors control for any post-intervention variables that could have been affected by the intervention? |  | NA / Y / PY / PN / N / NI |
| **Questions relating to baseline and time-varying confounding** |  |  |
| 1.7. Did the authors use an appropriate analysis method that controlled for all the important confounding domains and for time-varying confounding? |  | NA / Y / PY / PN / N / NI |
| 1.8. **If Y/PY to 1.7**: Were confounding domains that were controlled for measured validly and reliably by the variables available in this study? |  | NA / Y / PY / PN / N / NI |
| **Risk of bias judgement** |  | Low / Moderate / Serious / Critical / NI |
| Optional: What is the predicted direction of bias due to confounding? |  | Favours experimental / Favours comparator / Unpredictable |

| **Bias in selection of participants into the study** | | |
| --- | --- | --- |
| 2.1. Was selection of participants into the study (or into the analysis) based on participant characteristics observed after the start of intervention?  **If N/PN to 2.1:** go to 2.4 |  | Y / PY / PN / N / NI |
| 2.2. **If Y/PY to 2.1**: Were the post-intervention variables that influenced selection likely to be associated with intervention?  2.3 **If Y/PY to 2.2**: Were the post-intervention variables that influenced selection likely to be influenced by the outcome or a cause of the outcome? |  | NA / Y / PY / PN / N / NI  NA / Y / PY / PN / N / NI |
| 2.4. Do start of follow-up and start of intervention coincide for most participants? |  | Y / PY / PN / N / NI |
| 2.5. **If Y/PY to 2.2 and 2.3, or N/PN to 2.4**: Were adjustment techniques used that are likely to correct for the presence of selection biases? |  | NA / Y / PY / PN / N / NI |
| **Risk of bias judgement** |  | Low / Moderate / Serious / Critical / NI |
| Optional: What is the predicted direction of bias due to selection of participants into the study? |  | Favours experimental / Favours comparator / Towards null /Away from null / Unpredictable |

| **Bias in classification of interventions** | | |
| --- | --- | --- |
| 3.1 Were intervention groups clearly defined? |  | Y / PY / PN / N / NI |
| 3.2 Was the information used to define intervention groups recorded at the start of the intervention? |  | Y / PY / PN / N / NI |
| 3.3 Could classification of intervention status have been affected by knowledge of the outcome or risk of the outcome? |  | Y / PY / PN / N / NI |
| **Risk of bias judgement** |  | Low / Moderate / Serious / Critical / NI |
| Optional: What is the predicted direction of bias due to classification of interventions? |  | Favours experimental / Favours comparator / Towards null /Away from null / Unpredictable |

| **Bias due to deviations from intended interventions** | | |
| --- | --- | --- |
| **If your aim for this study is to assess the effect of assignment to intervention, answer questions 4.1 and 4.2** | |  |
| 4.1. Were there deviations from the intended intervention beyond what would be expected in usual practice? |  | Y / PY / PN / N / NI |
| 4.2. **If Y/PY to 4.1**: Were these deviations from intended intervention unbalanced between groups *and* likely to have affected the outcome? |  | NA / Y / PY / PN / N / NI |
| **If your aim for this study is to assess the effect of starting and adhering to intervention, answer questions 4.3 to 4.6** | |  |
| 4.3. Were important co-interventions balanced across intervention groups? |  | Y / PY / PN / N / NI |
| 4.4. Was the intervention implemented successfully for most participants? |  | Y / PY / PN / N / NI |
| 4.5. Did study participants adhere to the assigned intervention regimen? |  | Y / PY / PN / N / NI |
| 4.6. **If N/PN to 4.3, 4.4 or 4.5**: Was an appropriate analysis used to estimate the effect of starting and adhering to the intervention? |  | NA / Y / PY / PN / N / NI |
| **Risk of bias judgement** |  |  |
| Optional: What is the predicted direction of bias due to deviations from the intended interventions? |  |  |

| **Bias due to missing data** | | |
| --- | --- | --- |
| 5.1 Were outcome data available for all, or nearly all, participants? |  | Y / PY / PN / N / NI |
| 5.2 Were participants excluded due to missing data on intervention status? |  | Y / PY / PN / N / NI |
| 5.3 Were participants excluded due to missing data on other variables needed for the analysis? |  | Y / PY / PN / N / NI |
| 5.4 **If PN/N to 5.1, or Y/PY to 5.2 or 5.3**: Are the proportion of participants and reasons for missing data similar across interventions? |  | NA / Y / PY / PN / N / NI |
| 5.5 **If PN/N to 5.1, or Y/PY to 5.2 or 5.3**: Is there evidence that results were robust to the presence of missing data? |  | NA / Y / PY / PN / N / NI |
| **Risk of bias judgement** |  | Low / Moderate / Serious / Critical / NI |
| Optional: What is the predicted direction of bias due to missing data? |  | Favours experimental / Favours comparator / Towards null /Away from null / Unpredictable |

| **Bias in measurement of outcomes** | | |
| --- | --- | --- |
| 6.1 Could the outcome measure have been influenced by knowledge of the intervention received? |  | Y / PY / PN / N / NI |
| 6.2 Were outcome assessors aware of the intervention received by study participants? |  | Y / PY / PN / N / NI |
| 6.3 Were the methods of outcome assessment comparable across intervention groups? |  | Y / PY / PN / N / NI |
| 6.4 Were any systematic errors in measurement of the outcome related to intervention received? |  | Y / PY / PN / N / NI |
| **Risk of bias judgement** |  | Low / Moderate / Serious / Critical / NI |
| Optional: What is the predicted direction of bias due to measurement of outcomes? |  | Favours experimental / Favours comparator / Towards null /Away from null / Unpredictable |

| **Bias in selection of the reported result** | | |
| --- | --- | --- |
| Is the reported effect estimate likely to be selected, on the basis of the results, from... |  |  |
| 7.1. ... multiple outcome *measurements* within the outcome domain? |  | Y / PY / PN / N / NI |
| 7.2 ... multiple *analyses* of the intervention-outcome relationship? |  | Y / PY / PN / N / NI |
| 7.3 ... different *subgroups*? |  | Y / PY / PN / N / NI |
| **Risk of bias judgement** |  | Low / Moderate / Serious / Critical / NI |
| Optional: What is the predicted direction of bias due to selection of the reported result? |  | Favours experimental / Favours comparator / Towards null /Away from null / Unpredictable |

| **Overall bias** | | |
| --- | --- | --- |
| **Risk of bias judgement** |  | Low / Moderate / Serious / Critical / NI |
| Optional: What is the overall predicted direction of bias for this outcome? |  | Favours experimental / Favours comparator / Towards null /Away from null / Unpredictable |

**Section 10: Additional information**

**Withdrawal information:** *Please indicate frequency and cause of withdrawals.*

________________________________________________________________________________________________________________________________________________________________________________________________________________________

**General notes:**

________________________________________________________________________________________________________________________________________________________________________________________________________________________
